# Rotating Night Shift Work and Bladder Cancer Risk in Women: Results of Two Prospective Cohort Studies

**DOI:** 10.3390/ijerph20032202

**Published:** 2023-01-26

**Authors:** Shahab Haghayegh, Yue Liu, Yin Zhang, Susanne Strohmaier, Kyriaki Papantoniou, Sarah Markt, Edward Giovannucci, Eva Schernhammer

**Affiliations:** 1 Channing Division of Network Medicine, Brigham and Women’s Hospital and Harvard Medical School, Boston, MA 02115, USA; 2 Department of Epidemiology, Johns Hopkins Bloomberg School of Public Health, Baltimore, MD 21205, USA; 3 Department of Nutrition, Harvard TH Chan School of Public Health, Boston, MA 02115, USA; 4 Department of Epidemiology, Center for Public Health, Medical University of Vienna, 1090 Vienna, Austria; 5 Department of Population and Quantitative Health Sciences, Case Western Reserve University, Cleveland, OH 44106, USA; 6 Department of Epidemiology, Harvard TH Chan School of Public Health, Boston, MA 02115, USA

**Keywords:** shiftwork, cancer, circadian rhythm

## Abstract

Bladder cancer is the sixth most common cancer in the United States. Night shift work has previously been linked with cancer risk. Whether there is an association between rotating night shift work and bladder cancer in women has not been studied previously. Eligible participants in the Nurses’ Health Study (NHS, n = 82,147, 1988–2016) and Nurses’ Health Study II (NHSII, n = 113,630, 1989–2015) were prospectively followed and a total of 620 and 122 incident bladder cancer cases were documented during the follow-up of NHS and NHSII, respectively. Cox proportional hazards models were used to estimate hazard ratios (HR) and 95% confidence intervals (95% CI) for bladder cancer incidence. We observed a significantly increased risk of bladder cancer among women with >5 years of night shift work history compared with women who never worked rotating night shifts in NHS (HR = 1.24; 95%CI = 1.01–1.54, *p* for trend = 0.06), but not in the pooled NHS and NHS II (HR = 1.18; 95%CI = 0.97–1.43, *p* for trend = 0.08). Secondary analyses stratified by smoking status showed no significant interaction (*p* = 0.89) between the duration of rotating night shift work and smoking status. In conclusion, our results did not provide strong evidence for an association between rotating night shift work and bladder cancer risk.

## 1. Introduction

About 15–30% of the US and European workforce engages in shift work [1,2,3] in various industries such as healthcare, transportation, mining, and manufacturing [1,4]. The international agency for research on cancer (IARC) classified shift work as a probable human carcinogen (group 2A carcinogen) [1,5,6]. Several studies have demonstrated an association between shift work and various cancers, such as breast cancer [7,8,9,10], lung cancer [11], prostate cancer [12,13,14], colorectal cancer [15,16], and endometrial cancer [17]. 

Bladder cancer is the sixth most common cancer in the United States and accounts for 3% of global cancer diagnoses [18,19]. The incidence of bladder cancer increases with age, with the average age at diagnosis being 73 years, and is higher in men [19], while survival rates are lower in women [19,20]. Tobacco smoking (and environmental tobacco smoke) and environmental or occupational exposures are the most important known risk factors for bladder cancer [18,20,21]. Differences in the prevalence of tobacco smoking between men and women, greater environmental exposure in men, and hormonal differences may explain the gender disparity in bladder cancer incidents [18,20,22].

Few studies to date have examined the association between rotating night shift work and bladder cancer. These studies focused only on male participants and had small sample sizes [23,24], with both studies suggesting a significantly increased risk of bladder cancer among men who ever worked night shifts. In the current analysis, we used data from two large cohort studies, the Nurses’ Health Study (NHS) and Nurses’ Health Study II (NHSII), to examine the association between rotating night shift work and bladder cancer risk in women. We hypothesize that a longer duration of rotating night shift work is associated with an increased risk of bladder cancer.

## 2. Materials and Methods

### 2.1. Study Population

This study was performed utilizing the data from two large prospective cohort studies of US female nurses: the NHS and NHSII [25,26,27,28]. The NHS began in 1976, enrolling 121,701 participants aged 30 to 55 years, and NHSII began in 1989, enrolling 116,429 participants between 25 and 42 years old. In both cohorts, demographic data were collected at the baseline questionnaire. Information on anthropometrics, lifestyle, diet, medical history, and incident disease diagnoses were updated biennially or quadrennially via self-administered questionnaires throughout the follow-up. The response rate was more than 90% across questionnaire cycles. The Institutional Review Board of the Brigham and Women’s Hospital (Boston, MA, USA), and those of participating registries (as required), approved the study protocols. Informed consent was received from participants by the completion and return of the questionnaires. We used 1988 as the baseline for the NHS and 1989 for NHSII when rotating night shift work exposure was first assessed. Participants were excluded from analyses if they had no information on their age (NHS:172; NHSII:17), rotating night shift work history at baseline (NHS: 31,904; NHSII: 581), or died (NHS: 3571; NHSII: 0) or had any cancer diagnosed prior to the baseline (NHS: 3907; NHSII: 1043), leaving 82,147 eligible women in the NHS and 114,788 in NHSII.

### 2.2. Ascertainment of Rotating Night Shift Work

Total duration of rotating night shift work (defined as at least 3 nights/month, in addition to evenings and days) was assessed in 1988 in the NHS, with “never, 1–2, 3–5, 6–9, 10–14, 15–19, 20–29, and ≥30 years” as response categories [29,30]. Participants in NHSII were first queried about lifetime rotating night shift work history in 1989, with “never, 1–2, 3–5, 6–9, 10–14, 15–19, and ≥20 years” as response categories, and with regular updates thereafter [29,30]. In the final analyses, participants were classified into three categories (never, 1–5, and >5 years) according to their total duration of rotating night shift work.

### 2.3. Ascertainment of Bladder Cancer Cases and Participant Deaths

Information on physician-diagnosed incident cases of bladder cancer was reported biennially via questionnaires. With participants’ permission, cohort investigators accessed medical records and pathology reports or referred to state cancer registries (when medical records were unavailable) to ascertain diagnoses. We categorized tumors that had invaded subepithelial connective tissue, muscle, perivesical tissues, pelvic wall or abdominal wall, or metastasis (T1-T5) as invasive cancer cases, whereas stage Ta (Non-invasive papillary carcinomas) were considered as non-invasive cancer cases, and Carcinoma in situ (CIS) tumors were considered invasive due to high risk of progression [31,32]. Cohort investigators confirmed death events through routine searches of the National Death Index, next-of-kin reporting, or postal authorities with an identifying rate exceeding 98% [33,34].

### 2.4. Ascertainment of Covariates

Throughout the follow-up in both the NHS and NHSII, information on body weight, calculated BMI (kg/m2), smoking behavior (never, former, current; and calculated pack years, and time since quitting smoking), multivitamin use, history of diabetes mellitus, menopausal status, and postmenopausal hormone use were updated biennially. Information on every specific type of physical activity (based on which we calculated total physical activity in metabolic equivalent of tasks ((MET)-hours per week) and diet (e.g., alcohol, fruits and vegetables, bacon, total fluid, and total energy, assessed via semiquantitative food frequency questionnaires) were reported quadrennially. Information on race, height, and U.S. geographic region of residence (West, Midwest, South, Northeast) were assessed at baseline. The validity and reproducibility of information on covariates have been previously reported [35,36,37,38,39,40,41,42,43,44,45,46].

### 2.5. Statistical Analysis

We calculated person-years of follow-up from the return date of the baseline questionnaire (NHS: 1988; NHSII: 1989) to the date of bladder cancer diagnosis, date of death, or the end of the follow-up period (NHS: 30 June 2016; NHSII: 30 June 2015), whichever was earliest.

We performed separate analyses in each cohort, then conducted pooled analyses in the combined dataset. Using random-effects meta-analysis, we determined the *p*-value for heterogeneity across cohorts (*p* = 0.13). Cox proportional hazards models were used to estimate hazard ratios (HR) and 95% confidence intervals (95% CI) for bladder cancer incidence, given no evidence for violation of the proportional hazard assumption. *p*-values for trends were calculated using the mid-point values of rotating night shift work duration categories (never, 1–5, and >5 years) and modeling these values as a continuous variable.

Age-adjusted analyses were stratified by age (in months), cohort (in the pooled analyses), and questionnaire cycle (each at a two-year interval). Multivariable analyses were stratified by age, cohort, and questionnaire cycle, and additionally adjusted for a wide spectrum of established or suspected risk factors, including race (White, non-White), BMI (continuous, kg/m^2^), current smoking status and years since quit smoking (never, past smoker ≤5 years since quit smoking, past smoker 5–9 years since quit smoking, past smoker ≥10 years since quit smoking, past smoker unknown years since quit smoking, current smoker 1–14 cigs/day, current smoker 15–24 cigs/day, current smoker ≥25 cigs/day, and current smoker unknown cigs/day), pack-years of smoking (continuous, pack-years), alcohol intake (continuous, grams/day), physical activity (continuous, MET-hours/week), multivitamin use (yes, no), history of diabetes mellitus (yes, no), menopausal status (premenopausal or no history of postmenopausal hormone use, past menopausal hormone use, current postmenopausal hormone use), total fluid intake (continuous, mL/day), fruit and vegetable intake (continuous, servings/day), total calorie intake (continuous, kcal/day), bacon intake (continuous, servings/week), and U.S. geographic region of residence (West, Midwest, South, Northeast). Since the updated exposure information was available for the NHS II, the models using the updated information on rotating night shift work history are also presented for this cohort. Additionally, we conducted stratified analyses by smoking status (never-, past or current light-, and past or current heavy-smoker) and follow-up time (first and second half) to evaluate whether these factors modified the association of interest. Tests for interactions were conducted by adding interaction terms to the models and using likelihood ratio tests to determine statistical significance.

In case of missing data, information was carried forward by one cycle from the latest valid data to minimize missing information of repeatedly measured variables. For the remaining missing categorical variables after replacement, missing indicators were created and included in the models when necessary.

We performed data analyses using SAS statistical software (version 9.4 for UNIX; SAS Institute, Inc., Cary, NC, USA). All tests were two-sided with *p* values <0.05 indicating statistical significance.

## 3. Results

Table 1 presents the pooled baseline characteristics of participants in NHS and NHSII. Participant characteristics of NHS and NHSII at baseline are summarized in Tables S1 and S2. Overall, 39% of the participants had no history of rotating night shift work and 16% had worked rotating night shift work for >5 years. Briefly, the age of participants at the baseline in NHSII was ~20 years younger than NHS, and they most commonly lived in Midwest U.S., while in NHS, participants mostly lived in the Northeast U.S. Compared to those who had never worked rotating night shifts, participants with a longer duration of shift work had higher BMI, were more likely to be a smoker and physically active, have a history of diabetes mellitus, consume less alcohol, more fruit and vegetable, and consume more calories. During the follow-up, 742 cases of bladder cancer (620 in NHS, 122 in NHSII) were documented.

Table 2 shows the association between the duration of rotating night shift work and bladder cancer in NHS, NHSII, and pooling NHS and NHSII. There was no significant increase in the risk of bladder cancer with increasing years of rotating night shift work (for women with >5 years of rotating night shift work history vs. women who never worked in pooled NHS and NHSII, multivariable-adjusted HR = 1.18; 95% CI= 0.97 to 1.43, and *p*_trend = 0.08). There were no substantial differences in the findings of these analyses using only baseline covariates data rather than updating them during follow-ups (data not shown).

Table 3 presents the relative risk of bladder cancer by the duration of night shift work stratified by smoking status in the pooled NHS and NHS II. The results show no significant association between bladder cancer and rotating night shift work duration among any of the stratified smoking status groups (*p*_trend for never smokers, light smokers, and heavy smokers was 0.49, 0.74, and 0.09, respectively).

In another secondary analysis presented in Table S3, we stratified the association of bladder cancer risk and rotating night shift work duration by follow-up period (first half vs. second half of the entire follow-up duration). There was a significant trend in the association of bladder cancer risk and rotating night shift work duration when restricting the follow-up to the first half (*p*_trend = 0.03). Additionally, for nurses with >5 years of rotating night shift work history, the risk of bladder cancer in the first half of follow-ups was slightly higher than in the second half of follow-ups (HR = 1.32; 95% CI = 0.97 to 1.78 vs. HR = 1.13; 95% CI = 0.86 to 1.48).

## 4. Discussion

To the best of our knowledge, this is the first prospective cohort study evaluating the association between rotating night shift work and bladder cancer risk in women. We found a modestly suggestive, though not statistically significant, increased risk of bladder cancer after more than 5 years of night shift work based on 742 incident bladder cancer cases. Our findings are in line with two previous studies on this topic, although those studies focused on male participants. In a cohort of male production workers in Germany (12,609 shift workers and 15,219 day workers), the standardized incidence ratio of bladder cancer was increased for shift workers, but not for day workers. However, because this study was conducted in a chemical company, possible concurrent exposure to chemicals may have contributed to an increased risk of bladder cancer [23]. In a case–control study conducted in Montreal, Canada, which included 3173 patients diagnosed with 11 of the most frequent cancers, the adjusted odds ratio for bladder cancer in men who had ever worked night shifts was 1.74 (95% CI: 1.22, 2.49) compared to men who never had worked at night [24].

The differences in findings between our two cohorts warrant an explanation. In the older cohort (NHS), most women were close to retirement at baseline, whereas in the younger cohort (NHSII), women continued to work throughout follow-up, allowing us to assess for differences in the effect of night work on bladder cancer both during and after completing night work. We show that in the first half of follow-ups in NHS, where women were presumably still working, the risk was higher than in the second half, i.e., after retirement, when the exposure stopped. The weaker association in the younger women of NHS2 might suggest that we did not have enough power (20 cases in the top category only) or that the effect of circadian disruption in the form of night work has a long latency period, and, therefore, it was more pronounced among the older women. Alternatively, the underlying cancer rate, which differs by age, might explain the differences in the effect estimates between the two cohorts.

Previous studies have shown that inflammation may play an important role in the etiology of bladder cancer [47]. Furthermore, hypertensive patients are at higher risk of bladder cancer [48]. Given that shift work is a risk factor for inflammation [49,50,51] and hypertension [52], they could provide a mechanistic link for the association between shift work and bladder cancer risk. Some other potential mechanisms linking shift work to cancer promotion include: physiological disturbances due to phase shift [53], circadian system disruption [54], suppression of melatonin due to nighttime light exposure [53,54], sleep disturbances [53,54], lifestyle changes [53], and lower vitamin D due to reduced sunshine exposure [53].

The strengths of this study are: (1) using the data of two large cohort studies with about 195,000 total participants and more than 700 cases of bladder cancers; (2) the long duration of follow-up in both cohorts (28 years of follow-up in NHS and 26 years of follow-up in NHSII); and (3) availability of detailed information on a large number of potential confounding variables such as diet, physical activity, and smoking status. However, this study also has some limitations: (1) information on years of shift work in NHS is based on a one-time data collection at baseline; (2) lack of information on the time-schedule of the shift work in these cohorts; (3) although we captured more than 700 cases of bladder cancer in the follow-up duration, power was still limited in some categories, such as rotating night shift work duration of >5 years; (4) the possibility of healthy worker bias; and (5) lack of any male participants in these cohorts.

## 5. Conclusions

In this study, we found a modest positive association between the duration of rotating night shift work and bladder cancer risk. This effect was stronger in the short-term period. However, additional large studies with male and female participants and detailed assessment of possible confounders, such as circadian rhythm disruption, are needed to further evaluate the association between rotating night shift work and bladder cancer.

## Figures and Tables

**Table 1:** Age and age-adjusted characteristics of 196,935 study participants according to their total duration of rotating night shift work in the pooled Nurses’ Health Study (baseline in 1988, n = 82,147) and Nurses’ Health Study II (baseline in 1989, n = 114,788).

Characteristic	Total Duration of Rotating Night Shift Work		
	Never (N = 76,748)	1 to 5 Years (N = 89,495)	>5 Years (N = 30,692)
Age, years, mean (SD)	43 (11.5)	41.8 (11.5)	45.9 (11.8)
White, %	97.0	96.2	95.6
BMI, kg/m^2^, mean (SD) ^a^	24.5 (4.8)	24.6 (4.9)	25.6 (5.5)
Physical activity, MET-hours/week, mean (SD) ^b^	19.3 (29.5)	21.6 (32.2)	23.0 (34.7)
Pack-years of smoking, mean (SD) ^c^	7.3 (13.7)	7.5 (13.6)	8.7 (14.9)
History of diabetes mellitus, %	1.1	1.1	1.8
Alcohol, g/day, mean (SD)	3.7 (7.7)	3.9 (7.8)	3.5 (7.3)
Multivitamin use, %	42.1	42.8	42.8
Fruits and vegetables intake, g/day, mean (SD)	4.5 (2.3)	4.6 (2.4)	4.7 (2.5)
Total fluid intake, ml/day, mean (SD)	1893 (675)	1920 (682)	1971 (707)
Total calorie intake, kcal/day, mean (SD)	1754 (480)	1779 (485)	1783 (496)
Bacon intake, serving/week, mean (SD)	0.5 (0.8)	0.5 (0.8)	0.5 (0.9)
Menopausal hormone use, %	16.9	17.3	17.1
U.S. Geographic Region, %			
West	18.0	18.5	17.8
Midwest	29.6	29.6	32.2
South	14.0	13.9	12.9
Northeast	38.3	38.0	37.1

Abbreviations: SD, standard deviation; BMI, body mass index; MET, metabolic equivalent task. ^a^ Calculated as weight in kilograms divided by height in meters squared. ^b^ Weekly energy expenditure in MET-hours/week from recreational and leisure-time physical activity. ^c^ Cumulative among ever smokers.

**Table 2:** Hazard ratios and 95% confidence intervals (CI) of bladder cancer risk according to total duration of rotating night shift work in the NHS, NHS II, and pooled study population.

	Total Duration of Rotating Night Shift Work			*p*-Trend ^c^
	Never	1 to 5 Years	>5 Years	
**NHS (baseline)**				
No. of cases (N = 620)	225	252	143	
No. of Person-Years (2,024,544)	824,308	830,211	370,025	
Age-adjusted ^a^	1 [Ref]	1.09 (0.91–1.31)	1.32 (1.07–1.63)	0.01
MV-adjusted ^b^	1 [Ref]	1.10 (0.91–1.31)	1.24 (1.01–1.54)	0.06
				
**NHS** **II (baseline)**				
No. of cases (N = 122)	54	48	20	
No. of Person-Years (2,951,433)	1,121,476	1,442,723	387,234	
Age-adjusted ^a^	1 [Ref]	0.70 (0.48–1.04)	0.98 (0.58–1.63)	0.91
MV-adjusted ^b^	1 [Ref]	0.69 (0.47–1.02)	0.91 (0.54–1.53)	0.91
				
**NHS** **II (updated)**				
No. of cases (N = 122)	43	2	77	
No. of Person-Years (2,951,433)	907,918	58,010	1,985,505	
Age-adjusted ^a^	1 [Ref]	0.79 (0.19–3.26)	0.81 (0.56–1.18)	0.29
MV-adjusted ^b^	1 [Ref]	0.75 (0.18–3.11)	0.77 (0.53–1.12)	0.18
				
**Pooled NHS and NHS** **II**				
No. of cases (N = 742)	279	300	163	
No. of Person-Years (4,975,977)	1,945785	2,272,934	757,258	
Age-adjusted ^a^	1 [Ref]	1.01 (0.86–1.19)	1.25 (1.03–1.52)	0.02
MV-adjusted ^b^	1 [Ref]	1.01 (0.85–1.19)	1.18 (0.97–1.43)	0.08

Abbreviations: MV, multivariate; CI, confidence interval. ^a^ Stratified by age, cohort (pooled analyses only), and follow-up cycle. ^b^ Stratified by age, cohort (pooled analyses only), and follow-up cycle; adjusted for race, BMI, current smoking status, pack-years of smoking, time since quitting smoking, alcohol intake, physical activity, multivitamin use, menopausal status, history of diabetes mellitus, total fluid intake, fruit and vegetable intake, total calorie intake, bacon intake, and geographic region. ^c^ *p*-value for trend was calculated using the mid-point of each category of rotating night shift work duration in years.

**Table 3:** Hazard ratios (HR) and 95% confidence intervals (CI) of bladder cancer risk according to the total duration of rotating night shift work in the pooled NHS and NHS II, stratified by baseline smoking status.

	Total Duration of Rotating Night Shift Work			*p*-Trend ^b^	*p* for Interaction ^c^
	Never	1 to 5 Years	>5 Years		
**Never smokers**					
No. of cases (N = 242)	89	108	45		0.81
Age-adjusted	1 [Ref]	1.13 (0.85–1.51)	1.16 (0.80–1.68)	0.49	
MV-adjusted ^a^	1 [Ref]	1.13 (0.85–1.51)	1.16 (0.80–1.69)	0.49	
**Light smokers (<25 pack-years)**					
No. of cases (N = 203)	73	95	35		
Age-adjusted	1 [Ref]	1.14 (0.86–1.51)	1.12 (0.77–1.63)	0.64	
MV-adjusted ^a^	1 [Ref]	1.14 (0.85–1.52)	1.10 (0.75–1.60)	0.74	
**Heavy smokers (>=25 pack-years)**					
No. of cases (N = 297)	117	97	83		
Age-adjusted	1 [Ref]	0.77 (0.58–1.03)	1.18 (0.88–1.59)	0.09	
MV-adjusted ^a^	1 [Ref]	0.75 (0.56–1.00)	1.18 (0.87–1.59)	0.08	

Abbreviations: MV, multivariate; H.R., hazard ratio; CI, confidence interval. ^a^ Stratified by age, cohort, and follow-up cycle; adjusted for race, BMI, current smoking status (ever smoker only), time since quit smoking (ever smoker only), alcohol intake, physical activity, multivitamin use, menopausal status, history of diabetes mellitus, total fluid intake, fruit and vegetable intake, total calorie intake, bacon intake, and geographic region. ^b^ *p*-value for trend was calculated using the mid-point of each category of rotating night shift work duration in years. ^c^ Likelihood ratio test was used to calculate the *p* for interaction.

## Data Availability

Due to participant confidentiality and privacy concerns, data are available upon reasonable written request. According to standard controlled access procedure, applications to use NHS/NHSII resources will be reviewed by our External Collaborators Committee for scientific aims, evaluation of the fit of the data for the proposed methodology, and verification that the proposed use meets the guidelines of the Ethics and Governance Framework and the consent that was provided by the participants. Investigators wishing to use NHS/NHSII data are asked to submit a brief description of the proposed project (go to https://www.nurseshealthstudy.org/researchers (contact email: nhsaccess@channing.harvard.edu) for details.

## Supplementary Materials

The following supporting information can be downloaded at: https://www.mdpi.com/article/10.3390/ijerph20032202/s1, Table S1: Baseline characteristics of study participants according to the total duration of rotating night shift work in the NHS (baseline in 1988, n = 82,147); Table S2: Baseline characteristics of study participants according to the total duration of rotating night shift work in the NHS II (baseline in 1989, n = 114,788); Table S3: Hazard ratios (HR) and 95% confidence intervals (CI) of bladder cancer risk according to the total duration of rotating night shift work in the NHS and NHS II and stratified by follow-up time.
