# Supplementary material for: Rotating Night Shift Work and Bladder Cancer Risk in Women: Results of Two Prospective Cohort Studies
Source: Int J Environ Res Public Health. 2023 Jan 26;20(3):2202. doi: 10.3390/ijerph20032202 (PMC9915636; doi:10.3390/ijerph20032202)
Supplement: Supplementary file 1 [file ijerph-20-02202-s001.zip › Table S1.pdf]

Table S1. Baseline characteristics of study participants according to the total duration of rotating night shift work in the NHS (baseline in 1988, n=82,147)

Characteristic	Total Duration of Rotating Night Shiftwork		
	**Never** (N=33,142)	**1 to 5 Years** (N=33,417)	**>5 Years** (N=15,588)
Age, years, mean (SD)	54.4 (7.2)	54.7 (7.1)	56.1 (7.0)
Caucasian, %	97.9	97.7	97.0
BMI, kg/m^2, mean (SD) [a]	25.3 (4.6)	25.4 (4.6)	26.3 (5.2)
Physical activity, MET-hours/week, mean (SD) [b]	14.5 (20.6)	15.9 (21.7)	16.3 (22.8)
Pack-years of smoking, mean (SD) [c]	12.3 (18.3)	12.5 (18.2)	14.5 (19.8)
History of diabetes mellitus, %	2.7	2.5	4.4
Alcohol, g/day, mean (SD)	5.3 (9.7)	5.5 (9.8)	4.7 (9.3)
Multivitamin use, %	38.1	39.0	38.9
Fruit and vegetables intake, g/day, mean (SD)	5.2 (2.4)	5.3 (2.5)	5.4 (2.6)
Total fluid intake, ml/day, mean (SD)	2000 (629)	2027 (635)	2079 (650)
Total calorie intake, kcal/day, mean (SD)	1739 (461)	1768 (466)	1767 (479)
Bacon intake, serving/week, mean (SD)	0.6 (0.9)	0.6 (0.9)	0.7 (1.0)
Menopausal hormone use, %	37.6	38.5	37.7
U.S. Geographic Region, %			
West	13.7	12.3	10.4
Midwest	19.6	19.0	19.1
South	10.4	10.8	10.9
Northeast	56.3	57.9	59.6

Abbreviations: NHS, Nurses' Health Study; S.D., standard deviation; BMI, body mass index; MET, metabolic equivalent task.

[a] Calculated as weight in kilograms divided by height in meters squared.

[b] Weekly energy expenditure in MET-hours/week from recreational and leisure-time physical activity.

[c] Cumulative among ever smokers.
