# Supplementary material for: Rotating Night Shift Work and Bladder Cancer Risk in Women: Results of Two Prospective Cohort Studies
Source: Int J Environ Res Public Health. 2023 Jan 26;20(3):2202. doi: 10.3390/ijerph20032202 (PMC9915636; doi:10.3390/ijerph20032202)
Supplement: Supplementary file 1 [file ijerph-20-02202-s001.zip › Table S2.pdf]

Table S2. Baseline characteristics of study participants according to the total duration of rotating night shift work in the NHS II (baseline in 1989, n=114,788)

**Characteristic**	**Total Duration of Rotating Night Shiftwork**		
	**Never** (N=43,606)	**1 to 5 Years** (N=56,078)	**>5 Years** (N=15,104)
Age, years, mean (SD)	34.4 (4.7)	34.1 (4.7)	35.4 (4.0)
Caucasian, %	96.4	95.2	94.6
BMI, kg/m^2, mean (SD) [a]	23.9 (4.9)	24.0 (5.0)	25.0 (5.7)
Physical activity, MET-hours/week, mean (S.D.) [b]	22.7 (34.2)	25.7 (37.5)	27.7 (40.4)
Pack-years of smoking, mean (S.D.) [c]	3.7 (7.1)	3.9 (7.2)	4.6 (7.8)
History of diabetes mellitus, %	0.0	0.0	0.0
Alcohol, g/day, mean (SD)	2.6 (5.5)	2.8 (5.6)	2.6 (5.3)
Multivitamin use, %	45.0	45.6	45.5
Fruit and vegetables intake, g/day, mean (SD)	4.0 (2.1)	4.1 (2.2)	4.2 (2.3)
Total fluid intake, ml/day, mean (SD)	1816 (696)	1844 (704)	1895 (735)
Total calorie intake, kcal/day, mean (SD)	1764 (492)	1787 (497)	1794 (508)
Bacon intake, serving/week, mean (S.D.)	0.4 (0.6)	0.4 (0.7)	0.4 (0.7)
Menopausal hormone use, %	2.0	2.1	2.4
U.S. Geographic Region, %			
West	21.2	22.9	23.0
Midwest	36.9	37.1	41.4
South	16.7	16.1	14.3
Northeast	25.2	23.9	21.2

Abbreviations: NHS II, Nurses' Health Study II; S.D., standard deviation; BMI, body mass index; MET, metabolic equivalent task.

[a] Calculated as weight in kilograms divided by height in meters squared.

[b] Weekly energy expenditure in MET-hours/week from recreational and leisure-time physical activity.

[c] Cumulative among ever smokers.
