# Supplementary material for: Rotating Night Shift Work and Bladder Cancer Risk in Women: Results of Two Prospective Cohort Studies
Source: Int J Environ Res Public Health. 2023 Jan 26;20(3):2202. doi: 10.3390/ijerph20032202 (PMC9915636; doi:10.3390/ijerph20032202)
Supplement: Supplementary file 1 [file ijerph-20-02202-s001.zip › Table S3.pdf]

Table S3. Hazard ratios (HR) and 95% confidence intervals (CI) of bladder cancer risk according to the total duration of rotating night shift work in the NHS and NHS II and stratified by follow-up time

	Total Duration of Rotating Night Shift Work			*P*-trend [c]
	Never	1 to 5 years	>5 years	
**NHS**				
**Restricting follow-up to the first half (1988-2002)**				
No. of cases (N=247)	86	93	68	
Age-adjusted [a]	1 [Ref]	1.04 (0.77-1.40)	1.51 (1.10-2.08)	0.007
MV-adjusted [b]	1	1.04 (0.77-1.40)	1.39 (1.00-1.92)	0.03
**Restricting follow-up to the second half (2002-2016)**				
No. of cases (N=335)	126	138	71	
Age-adjusted [a]	1	1.09 (0.86-1.40)	1.27 (0.95-1.70)	0.13
MV-adjusted [b]	1	1.10 (0.86-1.40)	1.23 (0.91-1.65)	0.21
**NHS II**				
**Restricting follow-up to the first half (1989-2001)**				
No. of cases (N=33)	17	9	7	
Age-adjusted [a]	1	0.41 (0.18-0.93)	1.06 (0.44-2.56)	0.66
MV-adjusted [b]	1	0.41 (0.18-0.91)	0.97 (0.40-2.37)	0.79
**Restricting follow-up to the second half (2001-2015)**				
No. of cases (N=85)	34	40	11	
Age-adjusted [a]	1	0.93 (0.59-1.47)	0.87 (0.44-1.71)	0.69
MV-adjusted [b]	1	0.92 (0.58-1.45)	0.81 (0.41-1.62)	0.57
**Pooled**				
**Restricting follow-up to the first half**				
No. of cases (N=280)	103	102	75	
Age-adjusted [a]	1	0.93 (0.70-1.22)	1.43 (1.06-1.93)	0.005
MV-adjusted [b]	1	0.92 (0.70-1.21)	1.32 (0.97-1.78)	0.03
**Restricting follow-up to the second half**				
No. of cases (N=420)	168	178	82	
Age-adjusted [a]	1	1.06 (0.85-1.31)	1.19 (0.91-1.56)	0.21
MV-adjusted [b]	1	1.05 (0.85-1.30)	1.13 (0.86-1.48)	0.40

Abbreviations: MV, multivariate; H.R., hazard ratio; CI, confidence interval.

[a] Stratified by age, cohort (in the pooled analyses only), and follow-up cycle.

[b] Stratified by age, cohort (in the pooled analyses only), and follow-up cycle; adjusted for race, BMI, current smoking status, pack-years of smoking, time since quit smoking, alcohol intake, physical activity, multivitamin use, menopausal status, history of diabetes mellitus, total fluid intake, fruit and vegetable intake, total calorie intake, bacon intake, and geographic region.
